# Utilizing spiritual intelligence and workplace spirituality in creating collective awareness: the U-journey perspective

**DOI:** 10.3389/fpsyg.2024.1359562

**Published:** 2024-05-30

**Authors:** Elif Baykal

**Affiliations:** ^1^Business Administration Department, School of Business and Management Sciences, İstanbul Medipol University, İstanbul, Türkiye; ^2^Research Center of Innovative Management, Azerbaijan State University of Economics (UNEC), Bakü, Azerbaijan

**Keywords:** workplace spirituality, spiritual intelligence, U-journey perspective, collective awareness, change management

## Abstract

Theory U is a process-driven, learning, progress-directed, transformative, and relational approach to social change. This approach is predicated on the idea that spirituality may be used to create communal consciousness through change management. Dealing with spiritual hurdles, practicing meditation, improving sensing, staying in flow, and conceiving are just a few of the special skills needed for success on the U-journey. Spiritual intelligence also includes adaptive problem solving and goal achievement approaches. Theory-U holds that sources other than the outmoded paradigms that gave rise to complex problems are where answers to them must come from. The purpose of this paper is to demonstrate how individuals exposed to workplace spirituality can make better use of their spiritual intelligence. By using spiritual intelligence, people can attain the kind of awareness and engagement required for collective awareness, and this makes sense when we examine awareness awakening processes from the perspective of the U-journey.

## 1 Introduction

The practice of discernment is a significant tool for making the best decision about the future of an organization, one’s career, or new opportunities extends beyond traditional cognitive, social, and emotional processes. This is demonstrated by the rising interest in stress management, spiritual intelligence, workplace spirituality, and meaning-seeking (Nullens, 2019). In this regard, spiritual intelligence has finally been acknowledged as a valid intellectual ability according to Otto Scharmer and Peter Senge’s Theory U research at MIT (Scharmer, 2009). Through the lens of Otto Scharmer’s U Journey framework, a change management model, it becomes clear how important spiritual intelligence and workplace spirituality can be n fostering collective self-awareness (2009). It focuses on the process of becoming aware and is applicable to all stages of system transformation. Theory U offers sophisticated organizational learning and systems thinking tools that move us toward an awareness-based concept of systems change that integrates systems thinking and systems sensing. This approach blends systems thinking from the standpoint of evolving human awareness with leadership transformation.

In this paper, presumptions were derived from spirituality literature. A significant part of spirituality, both personal and professional, is the pursuit of meaning. According to Narcıkara (2018), spirituality is a psychological pattern that combines sentiments of integrity, meaningfulness, and connectedness to lead a more successful life. According to Baykal (2020), it is a state of awareness that promotes connectedness to both the internal and external worlds. Workplace spirituality, as defined by Jurkiewicz and Giacalone (2004), is derived from this literature and is a framework of values and corporate culture that represents a satisfying inner life, transparency, spiritual care, dependability, compassion, gratitude, and transcendence from the perspective of the organization. Workplace spirituality is independent of religious beliefs and, as it is based only on humanistic principles (Jnaneswar and Sulphey, 2021), it can promote harmony between the workplace and spirituality by highlighting the importance of both individual and communal spiritual intelligence. Spiritual intelligence must be mastered in order to comprehend the importance of spirituality on both a personal and a societal level. Spiritual intelligence (SI) is the capacity to use, manifest, and embody spiritual resources, values, and traits for the purpose of enhancing one’s well-being, according to Amram and Dryer (2008). SI was later defined by Griffiths et al. (2008) as a higher level of intellect that unlocks the genuine self’s potential for knowledge, compassion, and peace.

Vaughan (2003) asserts that spiritual intelligence is a prime example of spirituality’s adaptive role because it highlights spirituality’s capacity to make the connection between the inner spirit and mental condition and the outside world. It is closely related to the purpose of life, flexibility, spiritual resources, and external connectivity (Liu et al., 2021). Emotional and cognitive intelligence, for example, are built upon the foundation of spiritual intelligence. This position is filled by spiritual intelligence, which generates creative thinking and a holistic viewpoint in addition to offering spiritual advice and inspiration (Zohar and Marshall, 2004). One characteristic that sets spiritual intelligence apart is its focus on transcendental connection. Furthermore, according to Moghaddampour and Karimian (2013), people with strong spiritual connections are more willing to address moral and spiritual issues including awareness, justice, and respect for others. Since knowledge of life events and experiences accelerates the development of spiritual intelligence, people with high spiritual intelligence are more resilient to stress and can adjust to changing situations quickly (Liu et al., 2021). Spiritual intelligence has been crucial in helping people find answers to questions about their value and purpose in life, which has raised awareness on both a personal and a societal level (Liu et al., 2021).

In Scharmer’s (2009) U-journey, we enable ourselves to connect to the world outside of institutional bubble when we move down one side of the U, to connect to the world that arises from within when we move down the other side of the U, and to bring forth the new into the world when we move up the other side of the U. There’s an inner gate at the base of the U where we have to let go of anything that is not necessary. This method creates a subtle link to a more profound source of knowing by letting go of the old ego and present self and letting come of the best future self, highest future possibilities. Meeting at the bottom of the U and starting to listen to and resonate with each other are the fundamental aspects of presencing. These two selves are the best future selves and the present selves. Nothing stays the same after a group crosses this narrow line. Both the group and its individual members start to function with increased awareness and vitality and they start to sense possibilities for the future. They then frequently start to serve as a deliberate vehicle for a developing future.

In this paper, it is assumed that considering U-journey as a framework, spiritual intelligence of leaders and workplace spirituality in organizational culture can be utilized to create the necessary playground for success in creating collective self-awareness. Stated differently, the research question in this study is understanding whether the U-journey perspective can serve as a foundation for fostering a sense of collective consciousness within firms that use the spiritual intelligence of individual workers, which can be enhanced through workplace spirituality. With the U-journey template in hand, we will thus attempt to comprehend the function that spiritual-intelligence plays at the individual level and workplace spirituality plays at the organizational level in order to explain the development of collective self-awareness among subordinates.

## 2 U-journey perspective

The U-journey method developed by Scharmer (2009) is used in this study to promote both group consciousness and individual spiritual intelligence. It’s also a smart place to start when considering how businesses and organizations respond to significant events and crises that necessitate changes. Scharmer’s U Theory defies the traditional wisdom of starting from the past and creating a future that is equal to that past by thoroughly examining reality in an attempt to attain a blind spot (Scharmer, 2008). The U-journey requires going deeper into study and investigation in order to break free from the chains of routine, the past, ingrained views, and prejudices. It is at this point that individuals find true self-consciousness, which gives us the strength to climb back up, drawn by a future that is unlike anything that is left behind in the past.

The U-journey states that in order for a group or organization to successfully navigate Theory U, new collective consciousness must be developed. This can happen as a result of seven crucial leadership capacities: (1) Holding the space: allowing others to enter while being aware of what life is calling you to accomplish. (2) Observing: Silence your critical voice and maintain an open mind. (3) Presencing: connecting to the deepest source of yourself and your will with an open heart that allows you to see a situation from the whole and an open will that allows you to start acting from the emerging whole. (4) Sensing: connecting with your heart and cultivating your ability to appreciate and love. (5) Crystallizing: making use of one’s resolve. When a dedicated core group ventures out into the world with the goal of drawing people, opportunities, and resources, this capability is realized. (6) Prototyping: deliberately reintegrating the hand and heart intelligence: climbing the right side of the U requires deliberately reintegrating the head and heart intelligence; and (7) Performing: playing the macro violin. The violin metaphor references to playing the violin in a cathedral built to amplify sound at a macro level and illustrates the capacity to act and function from a bigger total.

Theory U provides answers from the perspective of human consciousness development by combining creativity, systemic thinking, and a change roadmap. The elements of Theory U consist of a new narrative for a societal evolution, a method for executing awareness-based change, and a framework for recognizing blind spots in leadership and system transformation. According to Scharmer (2004), a new type of collective leadership is required in order to overcome the threshold in difficult circumstances. This new form of leadership entails developing the ability to change the inside (the source). Regarding Scharmer (2004), effective leaders always reorganize the emphasis and structure of the group’s attention. This is what makes them successful. Rearranging an organization’s attentional structure can have the same beneficial effects as meditation, which increases awareness and broadens one’s potential responses to a given circumstance. Leadership assists organization in altering the composition of group attention, much as it does in mediation. This study assumes that workplace spirituality can be used by leaders to promote a feeling of community and function as a catalyst for the utilization of each employee’s own level of spiritual intelligence. This is the art and practice of leadership.

## 3 Spirituality

According to Baykal (2019a,b), spirituality in the workplace is about fostering a sense of community, advocating for social justice, and upholding the rights of all stakeholders to equitable representation and voice. It is the process through which individuals come to understand the importance of focusing their life on something bigger, more transcendent, and nonmaterial than themselves (2019). According to Giacalone and Jurkiewicz (2003), spirituality can be a useful instrument in satisfying the demands of workers who have an inbuilt yearning to find purpose in their profession and to be a part of a meaningful community that makes them feel valued and appreciated. Maslow’s beliefs could be seen as an early modern return to considering the spiritual mind within the human experience when examining the role of spirituality in psychology (Sargeant and Yoxall, 2023). Maslow defined transcendence as the process of going from being the best version of oneself to becoming something bigger than oneself—that is, being spiritual or altruistic for the benefit of others. Attaining this state of development, wherein one’s meaning and existence encompass a degree of spirituality, is considered the highest state of human understanding.

On the one hand, spirituality and religiosity are commonly acknowledged as phenomena that merit inquiry and have the potential to be the means of transcending. In actuality, two factors decide whether transcendence is considered religious or spiritual: (a) the level of definiteness; and (b) the system of references employed. In fact, cognitive processes are often employed by spiritual meaning systems to understand the immanent in a transcending manner (Narcıkara and Zehir, 2016). These interpretive frameworks are often institutionalized (as in theology) in religions, supported by a religious community, and reinforced by group behaviors. While the present is indeed transcendentally interpreted, the reference system used to describe what has been experienced remains ambiguous (e.g., a higher power or an ethereal energy), may differ, and/or is not based on institutionalized traditions. In contrast, spirituality is characterized by a greater range of individual interpretation and greater ambiguity (Jeserich et al., 2023).

## 4 Workplace spirituality

Sulastini et al. (2023) define spirituality as a multifaceted concept that is concerned with making a connection with something meaningful that goes beyond a person’s everyday existence. The fundamental idea of workplace spirituality, according to Nawaz et al. (2024) is bringing one’s physical, intellectual, emotional, and transcendental selves to work. According to Baykal and Zehir (2018), it is a psychological pattern that blends wholeness, connectedness, and meaningful existence. Three characteristics are typically used to define workplace spirituality: a sense of community, meaningful work, and inner life (Chawla and Guda, 2013; Benefiel et al., 2014). In this context, a person’s sense of belonging is defined by “care, relatedness, mutual obligations, and loyalty” and pertains to how they interact with others at work, while meaning is defined as how they understand how their work contributes to the greater good of their community or society (Alshebami et al., 2023).

Workplace spirituality, according to Long and Driscoll (2015), draws inspiration from organizational discourses such as corporate social responsibility (CSR), human relations movement, diversity management, authority, and positive organizational scholarship (POS). It also bears the same limitations in order to advance concepts of teamwork, involvement, and responsibility to others as fundamental objectives in and of themselves, going beyond being merely tasks for managers to complete.

## 5 Spiritual intelligence

Although many social scientists maintain that spirit is irrelevant and that intelligence is solely concerned with the mind, the terms “spiritual” and “intelligence” are intimately intertwined. A person’s level of happiness and contentment in life is influenced by “spiritual intelligence,” which is defined as the relationship between the spiritual and intelligence. It is the deep self’s intelligence, the intelligence of the soul. “The ability to act with wisdom and compassion while maintaining inner and outer peace, regardless of the circumstances” is the definition of spiritual intelligence given by Wigglesworth (2013). One with spiritual intelligence can reframe their reactions and pose basic questions (Srivastava, 2016). The literature now in publication offers a number of definitions for spiritual intelligence. Emmons (2000), for instance, states that “spiritual intelligence is a framework for recognizing and structuring the skills and abilities required for an adaptable utilization of spirituality.” Wolman (2001) defines spiritual intelligence as “the human capacity to ponder existential questions about the essence of life while simultaneously appreciating the flawless connection that exists between each of us and the wider environment in which we exist.” On the one hand, this corresponds to spiritual intelligence. “The capacity to draw on one’s spiritual gifts and assets to better recognize, find meaning in, and resolve fundamental, spiritual, and practical issues” is how Nasel (2024) later described spiritual intelligence.

Spiritual intelligence arises when consciousness deepens into a knowledge of matter, life, body, mind, soul, and spirit. Therefore, spiritual intelligence is more than just a person’s cognitive aptitude. It makes the assertion that it unites the ego and the spirit, as well as the transpersonal and personal (Srivastava, 2016). In accordance with, spirituality refers to a “set of mental capacities which contribute to the awareness, integration, and adaptive application of the nonmaterial and transcendent aspects of one’s existence, leading to such outcomes as deep existential reflection, enhancement of meaning, recognition of a transcendent self, and mastery of spiritual states”.

## 6 Methodology

Regardless of discipline, the foundation of all academic research activities is the development of research and its relationship to current knowledge. The field of business research is seeing a rapid acceleration of knowledge generation, which is transdisciplinary and fragmented at the same time. This makes it challenging to stay on the cutting edge of research, to stay current, and to evaluate the body of evidence in a certain field of study. For this reason, the literature review is more important than ever as a research methodology (Snyder, 2019). In this paper, an integrative review approach has been used since the purpose of the review is not to cover all articles ever published on the topic but rather to combine perspectives to create new theoretical relationships (Snyder, 2019). It is closely related to the semi-structured review approach but integrative review aims to evaluate, analyze, and synthesize the literature on a study issue in a way that makes room for the emergence of fresh theoretical frameworks and viewpoints (Torraco, 2005). Inclusion criteria for the articles that should take place in the review has been guided by the selected research question. In this paper, the research question was whether utilizing Spiritual Intelligence and Workplace Spirituality can be useful in Creating Collective Awareness. The U-Journey Perspective has been adopted as a change model in creating this organizational change. As a criteria for inclusion, we have chosen all related articles on U-journey approach, workplace spirituality and spirituality literature published in WOS, Scopus and Google Scholar in the last 15 years.

Actually, a pilot test of the review process covering the last 5 years was the initial stage in this study. By testing the search terms and inclusion criteria on a smaller sample it is attempted to see whether there are enough studies illuminating our research question. Since, studies regarding especially Theory-U was not enough we widened our scope to last 15 years. As to Snyder (2019), it is common to adjust this process a number of times before deciding upon the final time interval. Following the initial collection of articles, each one should be thoroughly checked to make sure it satisfies the inclusion requirements. As an additional tactic references of the chosen articles have also been examined to find more articles that might be of interest. After conducting the literature review, data abstracted are presented in the form of conceptualizations of the relationships mentioned in our research question in alignment with the purpose and research question of the review. When writing the review, we benefited from Torraco’s guidelines for integrative reviews (Torraco, 2005). As to Randolph (2019), when saturation is achieved and the reviewer has enough proof to persuade readers that every reasonable effort has been made to find all pertinent articles, the data gathering procedure can come to an end. Hence, in this study, literature review is finalized, after saturation point. Moreover, we ensured rigor and depth of the study by examining each assumed possible relationship in different parts and summarizing related literature in those parts. This also makes the study replicable by other researchers.

## 7 Spiritual intelligence and awareness

Knowing when and how to act with kindness and wisdom while upholding inner and outer harmony is a necessary component of spiritual intelligence (Aini et al., 2023). A person with a high level of spiritual intelligence is able to render fair and compassionate judgments. Aini et al. (2023) claim that spiritual intelligence is an indication of spiritual wellness. In this context, a state of spiritual health is characterized by acceptance, joy, ethics, and a sense of reciprocal connection with others, a sovereign and superior holy power, and oneself. It is described as a dynamic and well-coordinated process of cognitive, emotional, behavioral, and personal consequences. Spiritual knowledge, spiritual emotions, spiritual practices, and spiritual repercussions are some of the most significant facets of spiritual health.

Emmons (2000) defined spiritual intelligence as a person’s consciousness that encompasses the capacity to transcend and act morally, like showing greater humility and concern for others. In actuality, having a cerebral understanding of life’s purpose is associated with spiritual intelligence. It can be utilized to resolve conflicts among employees, leading to more cohesive business units and organizations, since it is a method for identifying, contemplating, and addressing spiritual concerns (Shateri et al., 2019). Noble (2001) established spiritual intelligence as a basic human potential by adding two more skills to Emmons’ (2000) list of necessary skills for spiritual intelligence.

1. The cognitive understanding that individuals engage, both consciously and unconsciously, on a moment-to-moment basis with a more expansive, multidimensional reality that is imbedded within physical reality. 2. The deliberate pursuit of psychological well-being for the benefit of oneself, the entire world community, and health communities and organizations is essential. Wigglesworth (2013) provided evidence to support the idea that spiritual intelligence upholds inner and exterior harmony by acting with wisdom and compassion and fostering higher states of consciousness.

According to Supriyanto et al. (2019), an individual possessing a higher level of spiritual intelligence may prove advantageous to the organization, as they may discover greater inspiration for their work. Spiritual intelligence is the capacity to use divine qualities to accomplish objectives and resolve problems, whereas spirituality is the experience of a higher consciousness and divine existence (Samul, 2020). One can, on the one hand, employ the awareness that comes from spiritual intelligence to cultivate a positive quality and make use of one’s talents to handle wrath and danger. Individuals that possess spiritual intelligence tend to be more genuine, compassionate, understanding, and mindful of others in their life (Vaughan, 2003).

## 8 Spiritual intelligence and collective awareness

According to McGhee and Grant (2017), spiritual intelligence can help reframe challenging work situations such that they align with the spiritual values and objectives of both individuals and organizations. When it comes to participatory approaches to spiritual intelligence, awareness “goes all the way down to the fundamental components of physicality,” perceiving mind and matter as being inextricably linked and the world as a dynamic, open-ended organism that is always engaged in co-creating itself (Zappalà, 2021). By integrating and synchronizing mental activity from all throughout the brain, including its physiological cues that increase attention, whole-brain reasoning generated by spiritual intelligence integrates the brain’s sequential and contextual systems (Zohar, 2016).

Walton (2017) claims that awareness has been “largely ignored in spirituality research” due to the “prevailing, often unconscious, influence of Newtonian science, which considers consciousness to be an epiphenomenon of the brain.” Walton also urges for the creation of research procedures that take into account the ways in which secular spiritual approaches that acknowledge the important connections between inner and outside experiences of consciousness are supported by developments in quantum physics. Because the observer is always contained within and a part of the observed reality, conscious observers and agents, or organizational members, co-create reality in organizational contexts (Zappalà, 2021).

In fact, we saw spiritual intelligence in this study as a kind of Complex Adaptive System (CAS), wherein the mind and environment interact to produce meaning and a high degree of consciousness (Zohar and Marshall, 2004). The ability to “access higher meanings, values, abiding purposes, and unconscious aspects of the self and to embed these meanings, values, and purposes in living a richer and more creative life” is the definition of spiritual intelligence given by Zohar and Marshall (2004) in reference to that point of view.

One of the essential traits of complex adaptive systems is their spontaneous organization, which frequently results in the self-assembly of a collective structure capable of methodically adapting to its surroundings through alertness, cunning, adaptability, and inventiveness (Zappalà, 2021). “Drivers” of the cognitive patterns within a common field of meaning and consciousness are the positive and negative motives (e.g., anger, fear, or mastery) that they see as driving behavior and thought patterns in complex adaptive systems. According to Zohar and Marshall (2004), spiritual intelligence, as a change intelligence, can assist people in moving from lower motivations, like anger, to higher motivations, like cooperation. The dynamics of change are usually brought about by the urge to adjust to an external crisis or to develop spiritual intelligences that may “pump energy into motivational states and redistribute energy to higher-energy motivational states” (Zohar and Marshall, 2004). We will talk about how this spiritual intelligence, or change intelligence, contributes to the development of collective self-awareness in the next section using the U-journey viewpoint, a crucial learning and change tool.

## 9 Creating collective awareness via U journey: utilizing spiritual intelligence

Theory U, a theory focused on transformation and problem-solving, was created by Otto Scharmer, Peter Senge, Joseph Jaworski, and Betty Sue Flowers (Sisk, 2016). Through more than 150 interviews, they identified a critical skill—presence—that is necessary to enter the field of the future with a high level of awareness. Two characteristics of the present, according to proponents of the U-journey, are the capacity for deep listening and the ability to look beyond one’s own perspective and conventional ways of making sense. There are four different ways to listen in the U-journey perspective: generative listening, which is the source of high-level awareness and the ability to connect to the best potential future, calls for you to access both your open heart and your open will. Everything that occurs throughout the process of verifying your preexisting beliefs is called “downloading.” Sound science is based on factual listening. The process of shifting from the it world of objects to the you-world, or the narrative of a living, changing person, is known as empathic listening.

On the one hand, leadership is essential in the U-journey method to altering the internal state from which we operate as individuals and as a group (Scharmer, 2009). Theoretically, Theory-U suggests that the solutions to the complex problems must come from sources other than the outdated paradigms that gave rise to them and we suggest that using spiritual intelligence on an individual basis and workplace spirituality on an organizational level are important new tools in this regard. Scharmer advocates for a shift in mindset and a novel approach to leadership (Nullens, 2019). Scharmer contends that we frequently watch the behaviors of leaders as well as the tactics and methods they employ. Scharmer (2016) observes that there appears to be a blind spot, or blindness to the inner place, the source from which effective leadership and social action emerge. Strange as it may sound, the outcome is ultimately determined by the leader’s inner place. Scharmer therefore thinks that in order to become more conscious, we must work and train more internally (Nullens, 2019). As to Scharmer (2008), collective blindness to the most profound aspects of transformational change and leadership is the root of collective failure. This “blind spot” is present in both daily social interactions and collective leadership. Individuals fail to see the underlying factor that gives rise to social action and good leadership. In U-journey methodology, effective leadership is predicated on the level of focus and intention a leader gives to any given circumstance (Scharmer, 2009). According to Scharmer (2009), in the process of driving transformational change, individuals rarely apply specific approaches to improve management performance from the inside out since they know so little about these interior aspects and they have problems in identifying and altering the structural attentional patterns that exist inside their businesses. This ignorance is similar to having a “blind spot” in the way people handle management and leadership. In order to lead effectively, we must first have a thorough understanding of the field, or inner space, in which we work. We must first comprehend the field, or inner space, from which we are functioning if we are to be good leaders. Four such “field structures of attention” are identified by Theory U, leading to four distinct modes of operation; 1. Operating from the old me world, 2.Operating from the current it world, 3.Operating from current-you world, 4. Operating from the highest future possibility that is wanting to emerge. As to Scharmer (2009), problems cannot be solved by the same level of consciousness that created them. So, leaders should create a collective consciousness in the field that the problems occur and transfer the organization to a new consciousness level, to a new field, if they want to make a profound change.

Not only do these disparate frameworks impact individuals’ listening skills, but they also have an impact on group communication and the way organizations establish their power hierarchies. Actually. As to Scharmer (2009), the course and manner in which a system takes are determined by the attention one pays to a given scenario. The single most significant leadership challenge of present day is to move from reactive answers and short solutions on a symptom level (Fields 1 and 2) to generative responses that address the systemic fundamental issues (Fields 3 and 4), which also necessitates mindfulness and high level of awareness which can be evoked by spirituality.

Otto Scharmer defined Theory U in terms of three things (Scharmer, 2009). It’s a framework, first and foremost, that begins with describing the process of transformation. Secondly, it serves as a tactic for bringing about change on a personal, collective, local, and global scale. Thirdly, it describes actual occurrences. Scharmer (2009) devised the U-journey as a technique to access more profound sources of awareness, which serve as the foundation for innovative ideas. Strong faith and trust are also important components of this process, which can all be improved on an individual basis by spiritual intelligence. Theory-U is an amazing model in the context of the broader shift toward post-materialist societal norms and increased consciousness, especially in light of the paradigmatic shifts it offers (Nullens, 2019). The Theory-U viewpoint states that when interpersonal levels of holistic communication modalities are employed and intrapersonal levels of cognitive, emotional, and physical resources are balanced, new dimensions of consciousness may emerge (Chlopczik, 2014). Creating a common attitude characterized by patience, humility, and mutual respect is the aim of this journey. By giving the person or a larger social system access to more profound information sources that are hidden from the downloading mind, the entire process seeks to raise consciousness.

Scharmer (2009, 70) makes a distinction between three types of knowledge on the one hand: There are three different kinds of knowledge: self-transcending knowledge, which is not yet embodied in the deep unconscious, implicit knowledge, which is already embodied but is concealed beneath the surface in a portion of the mind that is not fully conscious, and explicit knowledge, which is on the surface of people’s conscious minds. Organizations that aim to reduce self-consciousness at the community level should take seven crucial measures to initiate self-transcending knowledge. Put differently, there are seven steps in the developing process; the first is “downloading,” which is followed by “performing.”

The three fundamental motions that these seven U-journey phases represent are observe, retreat, reflect, and act (immediately). “Downloading” happens first in the observe-sequence, then “seeing” and “sensing.” Because it combines “presence” and “sensing,” Scharmer defined this process’s “presencing” moment as occurring during the retreat and reflect-movement. Most of our knowledge is derived from a cursory “downloading,” not from an intentional experience of the future as it unfolds. Scharmer actually offers a figurative learning theory in the form of a “U,” based on the three stages of attitudinal change that are “presencing,” “letting go,” and “letting arrive.” “Sensing” and “presence” are combined to generate the phrase “presencing.” “It entails being able to sense, tune in, and operate from one’s fullest future potential—the future that relies on us to bring it into being.” Regarding, people who go down the left side of the U.S. remain linked to the world outside of their cozy structures. He says that as one approaches the bottom of the U, one can connect with the world that arises inside of them. They’ve arrived at the “letting go” stage, which entails embracing and welcoming the new as they proceed up the right side of the U.

The field structure of attention and the state of consciousness are established through a process of refinement, according to Theory U (Chlopczik, 2014). Reaching the base of the U allows us to establish a connection with the inner world. As we move further up the right side of the U, we have arrived at the “letting go” stage, which is bringing forth and embodying the new. The movement of the U should be viewed as dimensions or a matrix of reality perception rather than as sequential stages (Scharmer, 2016, 44). There is an interior gate at the base of the U that compels travelers to abandon everything behind on that journey.

A delicate link to a deeper source of knowledge is established through the act of letting go (of the Self, the ultimate future possibility) and yielding (of the old ego and self). The fundamental concept of presencing is that at the bottom of the U, the best future version of oneself and the current version of oneself meet and start to resonate with each other. Observing and recording the future as it unfolds is one aspect of discernment. According to Theory U, the way you react to a situation affects how it develops (Sisk, 2016). The procedure involves having an open heart, open intellect, and open will. In fact, heightened spiritual intelligence can develop a spiritual mind that serves as a trigger for this process, leading to heightened attentiveness (Yilmaz et al., 2023). On this journey, taking action after retreating and letting go for a while is based on reflection. Nothing stays the same when someone or a group crosses this line. Individuals start to function more energetically and with a greater sense of possibilities for the future (Nullens, 2019).

Indeed, theory U takes a personal, individual-centered approach to dealing with the challenges of disruptive change that is also valid for spiritual intelligence (Fry and Wigglesworth, 2013). As to Emmons (2000), spiritual intelligence is the ability to experience heightened states of consciousness, hence it is meaningful to utilize spiritual intelligence in creating both individual and organizational level collective consciousness mentioned during the U-journey. Participants in this journey are encouraged to let go of preconceived notions about social interaction, which are frequently rooted in historical patterns, and to be receptive to fresh perspectives on how to live a more just, sustainable, and healthful lifestyle. Undoubtedly, this change requires a clear head as well as the transcendence of the human soul with higher purposes and meanings, which can be attained via spirituality and spiritual intelligence at the individual level (Watts and Dorobantu, 2023). Moreover, this shift has an effect on the social and economic spheres in addition to the individual (Heller, 2019). A four-phase model of linear historical progression is used by Theory U to shed light on the ramifications of that shift for society at large. According to Heller (2019), this model moves from the conventional authoritarian power structures that rule the economy and civil society to forms of cooperation driven by eco-system awareness. This model also fosters a collective awareness in which people learn about and become more conscious of all the relevant stakeholders and their expectations. Stated differently, collective awareness at the organizational and community levels necessitates a change in consciousness from the ego-system to the eco-system (Scharmer and Kaufer, 2013).

On the one hand, at this stage in the U-journey, one aspect of the spiritual dimension is particularly emphasized: reflective practice, which involves introspection and/or communication with a higher force (God, Allah, etc.). According to Nullens (2019), this is a method for improving management efficacy. This method can make use of spiritual intelligence since it gives people a more expansive view on life and reduces their dependence on their immediate environment (Ebrahimi et al., 2023). In actuality, spirituality is the outward expression of the innate ability of humans to transcend.

Narcıkara (2018) asserts that reestablishing one’s connection to one’s own set of values and integrating one’s life are fundamental to spiritual awareness. As previously said, the foundation of Theory-U’s social change model is the idea of purposefulness and collaboration, which leads to positive transformation and allows for the exploitation of this life integration and reconnecting. To put it another way, U-journey’s social change model, which consists of eight core values aimed at enhancing people’s capacity for cooperation and self-awareness, fosters collective self-awareness and makes the community more aware of the difficulties it faces both now and in the future (Sisk, 2016).

## 10 Discussion

Theory-U defines presencing as a state of enhanced awareness that enables individuals and organizations to change the internal environment in which they operate. It is a combination of the terms “presence” and “sensing.” According to Peschl and Fundneider (2014), it necessitates the development of completely new cognitive skills, attitudes, and epistemic virtues, such as intense openness, profound observation and understanding capacity, reframing, and so on. When that change happens, people begin to behave from a future space of potential that they sense can arise; this is referred to as “presencing.” That experience often sparks ideas for overcoming obstacles and achieving goals that do not seem possible. Theory U explains how to develop this kind of presence capacity.

People are bringing the new into the world when they move up one side of the U (bringing the new into the world), down one side of the U (connecting them to the world that arises from within), and along the other side of the U (connecting them to the world beyond of their institutional shell). They are forced to leave everything behind as they travel through an inner gate at the base of the U. A subtle link to a more profound source of knowledge is established via this process of letting go (of the old ego and self) and allowing arrive (of the highest future possibility: their Self). Organizations use this approach to deal with change most of the time.

Any functional system or structure is built around a well-established repertoire of reactions, such as routines, processes, or reflexes. It is dangerous to reduce the psychological repertoire to a relatively sophisticated system of responses because the ensuing remedies or changes are shallow and often barely address the surface of the real challenge that lies behind the change (Peschl and Fundneider, 2014). The reactions are also very rigid. A rearrangement and adaptation plan, on the other hand, goes a step farther and emphasizes the problem of rigidity and merely responding to changes. At this point, knowledge patterns that have already been discovered are used to solve the problem in addition to acting as a somewhat updated blueprint that has been modified to suit the particular situation (Peschl and Fundneider, 2014). One of the main issues that motivate this redesigning and redirecting level change-coping strategy is: How does one escape the frame of reference that traps them? This method’s goal is to examine one’s own perceptions and thought patterns in order to gain fresh perspectives that go beyond the limitations of a certain paradigm.

This procedure, called redirection, involves moving the center of attention from the exterior to the internal object. Consequently, one looks at the world through the lens of oneself and makes an effort to understand the cosmos from that perspective, which is also possible through spiritual intelligence. While this can be accomplished alone, social settings yield greater results and lead to a communal consciousness (Peschl and Fundneider, 2014). The reframing process takes this reflective process a step further, producing entirely new conceptual frameworks, fresh information, and archetypal spaces that enable the reframing of cognitive frameworks that are already generally accepted (Peschl and Fundneider, 2014).

## 11 Conclusion

According to Scharmer and Yukelson (2015), modifying systemic behaviors necessitates altering (deepening) the consciousness that every individual and group operating inside these systems brings to their conduct. This process of achieving these higher levels of understanding became known as Theory U (Scharmer, 2009). Theory U states that r = f (ai) indicates that the reality and outcomes (r) that a system of players enacts are determined by the awareness (a) from which the system of players functions. The quality of the results generated by a given system is determined by the participants’ level of awareness. Regarding Scharmer and Yukelson (2015), Theory U distinguishes between the four modes of awareness (also known as “field structures of attention”) that individuals, groups, institutions, and larger systems use on a regular basis. A “field” is defined as an assembly of related relationships. Every field condition of awareness has a unique inner cause, and workplace spirituality initiatives have the potential to greatly facilitate this transformation.

In actuality, theory U is a relational, transformative, process-oriented, learning, and change-directed paradigm of social change. Rather than being a single event, this journey consists of a sequence of actions that lead to a change in behaviors. Theory-U is described as “a coherent set of activities that are intentionally and regularly enacted by an individual” (Rothausen, 2017, 4), much like spirituality. Two significant terms in the Theory-U framework are “open mind” and “state of fundamental freedom.” Scharmer claims that presencing as a social technology is really a freedom technology (Scharmer, 2016, 184). Before we can co-create the new, we need to first make room in our minds for letting go of everything that is not essential. For this profound experience, we must find a sacred place for silence or deep listening, which is easily accomplished by a spiritually astute person. Silence facilitates interpersonal connections and sparks original creative thought during the U-Journey (Scharmer, 2016, 237). Researchers studying spirituality claim that by listening to one’s inner voice, spiritual intelligence may distinguish between what is “right” and “not right” within the context of a community or situation (Datta, 2021). Although most people ignore spiritual intelligence and miss out on a deeper feeling of being, people’s brains are hardwired to activate and utilize it (Singh and Sinha, 2013). Sisk (2016) asserts that spirituality encompasses a number of important elements that are applicable to U-journey, including fundamental abilities, resolving transcendental challenges, and engaging in sense, vision, and meditation exercises. Compassion, unity, and community are among the fundamental values. Peak experiences, transcendent emotions, and elevated consciousness are indications that one has understood the meaning of ultimate aspirations. Equality, truthfulness, compassion, and empathy are fundamental principles. Scharmer defines co-creation as a process that encompasses a wider range of innovative social and economic actions (Heller, 2019). Since there is a synergistic and larger degree of awareness when each person possesses a significant amount of spiritual intelligence, collective co-creation becomes more viable.

While there is not a clear cut road from spirituality to corporate culture transformation, spiritual intelligence is a concept that can be used as a tool in this process (McGhee and Grant, 2017). Differentiating between corporate and individual spirituality is crucial. Intrapersonal spiritual experiences have been the subject of individual investigations (Narcıkara and Zehir, 2016). This point of view makes the assumption that a person’s spirituality affects both their behavior and their interpretation and reaction to the work.

Similar to how “organizational culture” and “organizational strategy” are studied collectively, spirituality is also investigated collectively (McGhee and Grant, 2017). But common sense demands that every spiritual culture is the product of its members. Actually, if spirituality is the pursuit of meaning, then spiritual intelligence is a set of skills that one might employ in an organizational context to attain a more meaningful life (Steingard and Dufresne, 2013). According to Emmons (2000), there are numerous definitions of intelligence that involve adaptive problem solving and goal attainment using a set of special talents required for success in U-journey. Additionally, it may heighten peak experiences, flow states, and conscious awareness. On the one hand, a sense of community and unity is another aspect of spirituality. This sense is felt more strongly by organizational members when their individual level of spiritual intelligence is higher, and it leads to collective self-awareness and organizational transformation (Alshebami et al., 2023).

Moreover, the spiritual intelligence framework proposed by Emmons (2000) expands the definition of spirituality to encompass concepts that are not conventionally connected to it. For example, “Spiritual intelligence increases the plausibility of scientific spirituality by situating it within an already appropriate psychological system connecting personality and behavior.” It makes it possible for spirituality to be firmly rooted in logical strategies that prioritize achieving objectives and finding solutions to problems on both an individual and a group level. On the one hand, people’s spiritual intelligence can be used more skillfully when workplace spirituality is cultivated in corporate culture.

## 12 Managerial implications

The significance of spiritual intelligence at the individual level in advancing awareness and problem solving has started to garner attention since workplace spirituality has been on the agenda of organizational researchers as a source of organizational climate that can be a salve for modern organizations’ search for meaning and awareness. This study demonstrates the extent to which the relationship in question can aid in the development of a subjective awareness when viewed through the lens of the U-journey. Additionally, an attempt is made to elucidate how workplace spirituality facilitates the realization of the U-journey framework, which is centered around the pursuit of social change and cultivates spiritual intelligence at the individual level.

## Author contributions

EB: Conceptualization, Data curation, Formal analysis, Funding acquisition, Investigation, Methodology, Project administration, Resources, Software, Supervision, Validation, Visualization, Writing – original draft, Writing – review & editing.
